# Feeding the vasculature with cruciferous vegetables: a secret for organ protection

**DOI:** 10.1038/s41392-024-01747-x

**Published:** 2024-02-23

**Authors:** Priya Veluswamy, Jens Wippermann, Max Wacker

**Affiliations:** Heart Surgery Research, Department of Cardiothoracic Surgery, Otto-von-Guericke University Hospital, Magdeburg, Germany

**Keywords:** Molecular medicine, Inflammation, Cell biology

Two very recent back-to-back papers published in Nature^1,2^ decipher the holistic role of the aryl hydrocarbon receptor (AHR)/dietary-ligand axis, in enteric and pulmonary vasculature to emphasize tolerance induction and vessel integrity during homeostasis, sterile inflammation and infections. Both studies spectacularly highlight the significance of endothelium in orchestrating tissue tolerance by demonstrating the multifaceted activities of endothelial-expressed AHR that protects the vasculature to maintain organ homeostasis.

In general, AHR is a ligand-dependent cytosolic transcription factor that is traditionally known to generate responses to xenobiotic ligands (environmental pollutants, like dioxins). Of note, the endothelial AHR signals are also triggered by nutritional ligands, derived from cruciferous vegetables.

The excellent work reported by Wiggins and colleagues^1^ dissected the functional attributes of enteric endothelial AHR signaling in maintaining intestinal vascular normalcy, as they are invariably exposed to internal and external environmental cues. Prior to the knowledge acquisition on AHR signaling and outcome, the authors decoded the cellular complexity of murine enteric vasculature to first understand the heterogeneity of endothelial cell subtypes. These single cell resolution transcriptomic data revealed four lymphatic endothelial cells (LEC) and seven blood endothelial cell (BEC) clusters that were identified and grouped based on marker expressions and biological functions. Later, these classified enteric endothelial cell clusters were helpful to demonstrate their respective sensitivities to a tryptophan-derived AHR ligand (FICZ, 6-formylindolo [3, 2-b] carbazole), where a wide induction of the AHR-specific target gene, cytochrome P450 1A1 (CYP1A1), was observed in all endothelial cell subtypes. This underlines a global-sensing property of endothelial-expressed AHR towards AHR ligands. Though intracellular CYP1 enzymes are readily induced upon AHR activation, they act as negative regulators to curtail the duration of AHR signaling because of promoting metabolic clearance of AHR ligands.^3^ Besides, transcriptomic data disclosed increased expression of significant genes such as cyclin dependent kinase inhibitor 1A (*Cdkn1a*) (anti-proliferation), theoredoxin-interacting protein (*Txnip*) (oxidative stress protection), and krueppel-like factor 9 (*Klf9)* (cellular quiescence) and decreased expression of significant genes such as myristoylated alanine rich C-kinase substrate (*Marcks*) (endothelial cell motility), SRY-box transcription factor 18 *(Sox18)* (angiogenesis) and toll-like receptor 4 (TLR4)-interactor with leucine/rich repeats *(Tril)* (inflammatory signalling pathways) among these endothelial cell clusters that were exposed to AHR ligand. The authors considered this transcriptomic data as a blueprint to further investigate AHR-mediated signaling outcome among intestinal endothelial cells. This is achieved by generating an inducible endothelial cell-specific *Ahr*-deficient mouse model (EC^ΔAhr^), which certainly reinforces the concept that lack of AHR responsiveness to FICZ ligand enriched the pathways responsible for inflammation, angiogenesis, cell motility and leukocyte recruitment. All data were compared with *Ahr*-sufficient wild-type mice (EC^WT^). In EC^ΔAhr^ mouse gut, the authors showed increased BEC proliferation that is further amplified by adding an exogenous angiogenic factor, vascular endothelial growth factor A (VEGFA), showing plausible roles of AHR in limiting proliferation and restricting VEGFA signaling to curb angiogenesis. In fact, increased bioavailability of VEGFA among *Ahr*-deficit mice could be, in part, promoted by increased expression of the endothelial cell-specific molecule 1 (ESM1), a tip cell angiogenic marker that serves as a target for VEGFA.^4^

Next, the authors induced sterile inflammation using lipopolysaccharides (LPS) treatment in mouse with partial deletion of *Ahr* to affirm the transcriptomic findings that AHR deficiency increases gut endothelial inflammation. At the same time, they questioned whether the anti-inflammatory attributes of AHR impair protection against enteric pathogens. To answer this, they further challenged the EC^ΔAhr^ mouse with *Yersinia pseudotuberculosis* and noted that tissue tolerance was the contributing factor for significant improvement in survival of the *Ahr-*sufficient mouse. By translating the key findings to a human umbilical vein endothelial cell line (HUVECs) model, the authors validated that FICZ-triggered HUVECs exhibited an increased state of cellular quiescence with decreased proliferation rate.

In parallel, Major et al.^2^ focused on airways to demonstrate the beneficial effects of high AHR expression in endothelial cells during influenza virus infection. They demonstrated increased AHR activities in lung vascular endothelium in comparison to other lung-associated cells, including epithelium, type II alveolar cells and immune cells. Since CYP1 enzymes are negative regulators of AHR activation, the authors generated a *Cyp1*-deficit mouse to investigate uninterrupted expression and signaling of endothelial AHR during influenza infection. During the peak immune phase following infection (i.e. on day 6), mice with ablated *Cyp1* genes displayed increased vascular barrier resistance and improved survival rate. This mouse model also promoted protection against coinfection with *Streptococcus pneumoniae*, with decreased mortality and reduced clinical scores. In contrast, EC^ΔAhr^ mice exhibited influenza-induced loss of the vascular barrier resistance, with increased pulmonary inflammation and decreased survival. Here, a wide-range of lung-infiltrating immune cells, cytokine and chemokines, and serum albumin were set as measuring parameters to certify the lung vascular integrity. Importantly, a crosstalk between infected lung endothelium and epithelium was noticed that enriched the accumulation of apoptotic and necrotic epithelial cells, with keratinizing dysplasia in airways lacking AHR, signifying the role of endothelial-expressed AHR in preserving the functions of other cell types. Of note, the authors spotlighted the apelin (Apln) -apelin receptor (Aplnr) signaling system as the crucial pathway for AHR-mediated favorable outcome in lung protection.^2,5^ It was noted that EC^ΔAhr^ mice exhibited insufficiency of Aplnr expression. With this knowledge, the authors manipulated Aplnr by administering therapeutic Apln in wild-type and EC^ΔAhr^ mice and evidently found reduced lung damage and vascular leakage in wild-type mice. This concept was further strengthened by pharmacological blocking of Aplnr expression (MM54) in the *Cyp1*-deficit mouse that revealed the enrichment of endothelial cell stress response and increased vascular leakage, showing coordinated action of AHR and Aplnr in promoting lung vessel tolerance during influenza infection. When infection itself had dampened both AHR and Aplnr expressions, their activities were restored by supplementing with AHR ligand-rich diets, demonstrating the existence of a commendable tolerance-inducing mechanism at the gut-lung axis.

**Fig. 1:**
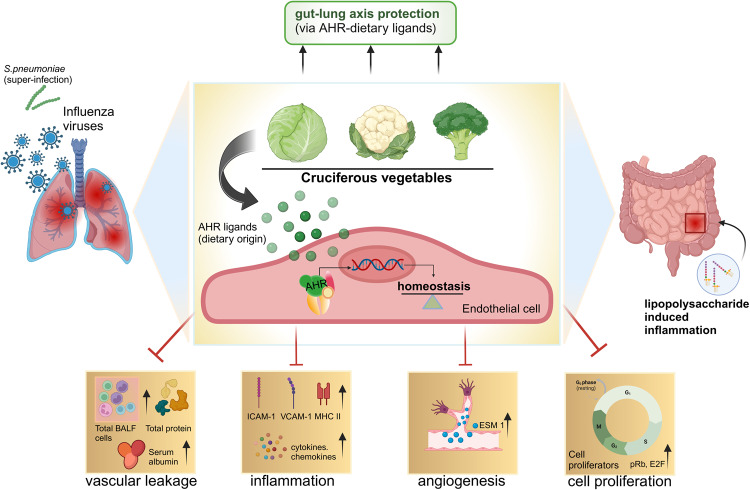
Multiple functions of AHR/dietary-ligand axis during infections and inflammation. Activation of cytosolic AHR in the endothelium by dietary ligands, derived from cruciferous vegetables like cabbage, broccoli and cauliflower, transcribe the genes responsible for maintaining tissue homeostasis. The endothelial AHR signaling improves the vascular barrier function by limiting the leakage of total cells, proteins and serum albumin into the lung lavage during influenza infection. This AHR-AHR ligand activation also remarkably protects against superinfection like *Streptococcus pneumoniae*. Furthermore, during inflammation, AHR signaling imparts protection against organ (gut) damage by increasing the state of tissue tolerance and cellular quiescence that are reflected by (i) decreased expression of inflammatory profiles, like intracellular adhesion molecules (ICAM-1), vascular cell adhesion molecules (VCAM-1), major histocompatibility complex (MHC-II) molecules and cytokines and chemokines; (ii) decreased expression of endothelial cell-specific molecule (ESM-1) to curb the process of angiogenesis; (iii) decreased expression of cell cycle proliferators, like retinoblastoma protein (pRb) and transcription factors in higher eukaryotes (E2F). These mechanistic attributes of the AHR/dietary-ligand axis contribute to a remarkable tissue tolerance on the gut-lung axis. This figure was created with BioRender.com

Collectively, the authors have zoomed into the endothelial cells to snapshot the activities of AHR on vessel barrier functions and tissue tolerance. Of note, the important homeostatic functions of AHR could be triggered by sufficient intake of AHR ligand rich foods to minimize organ damage during infection and inflammation (Fig.1). These ground-breaking works open several new channels for possible therapeutic options by (i) manipulating AHR receptors with nutritional ligands; (ii) triggering Apln-Aplnr axis; (iii) combinatorial therapies using nutritional ligands and agonistic apelin to induce endothelial AHR and Aplnr signaling, respectively, for improved organ homoeostasis.
